# Novel use of cutting balloon to manage compressive subintimal hematoma during left main stenting in a patient with spontaneous coronary artery dissection

**DOI:** 10.1002/ccr3.1531

**Published:** 2018-05-27

**Authors:** Brent M. McGrath, Minh N. Vo

**Affiliations:** ^1^ Division of Cardiology (Interventional Cardiology) New Brunswick Heart Centre Saint John Regional Hospital Saint John New Brunswick Canada; ^2^ Mazankowski Alberta Heart Institute University of Alberta Hospital Edmonton Alberta Canada

**Keywords:** Angioplasty, cutting balloon, spontaneous coronary artery dissection, subintimal hematoma

## Abstract

Spontaneous coronary artery dissection (SCAD) is a common cause of acute coronary syndrome particularly in younger women. Good outcomes with conservative management are generally expected. However, there is uncertainty of how to manage symptomatic or unstable patients. Percutaneous angioplasty may propagate the subintimal hematoma compromising coronary blood flow. Cutting balloon angioplasty can relieve the compressive effects of a propagated subintimal hematoma in SCAD.

## Introduction

Spontaneous coronary artery dissection (SCAD) is increasingly being recognized as an important and common cause of acute coronary syndrome. While the etiology and pathophysiology are not fully elucidated, SCAD appears to have a particular preponderance for younger women who are among the lowest risk of atherosclerotic heart disease. Due to the paucity of research data in this patient population, treatment strategies have largely been based on case reports, small cohort studies, and expert opinion.

Contemporary recommendations generally support good outcomes with conservative management in patients who are clinically stable 1, 2. However, there is uncertainty of how to manage patients with persistent symptoms or clinical instability. Revascularization may be necessary and appropriate in patients with ongoing or recurrent ischemia, ventricular arrhythmias, hemodynamic instability, and/or left main coronary artery dissection 3, 4.

Among patients with SCAD who undergo percutaneous coronary interventions (PCIs), short‐ and long‐term outcomes are unclear and propagation of subintimal hematoma has been reported in up to 57% of patients 5, 6. These compressive hematomas compromise antegrade coronary blood flow, and further stenting may cause a vicious cycle of further propagation and compression distally. Herein, we report a novel and elegant use of the cutting balloon to treat compressive subintimal hematoma following left main stenting in SCAD.

## Case History

**Figure 1:**
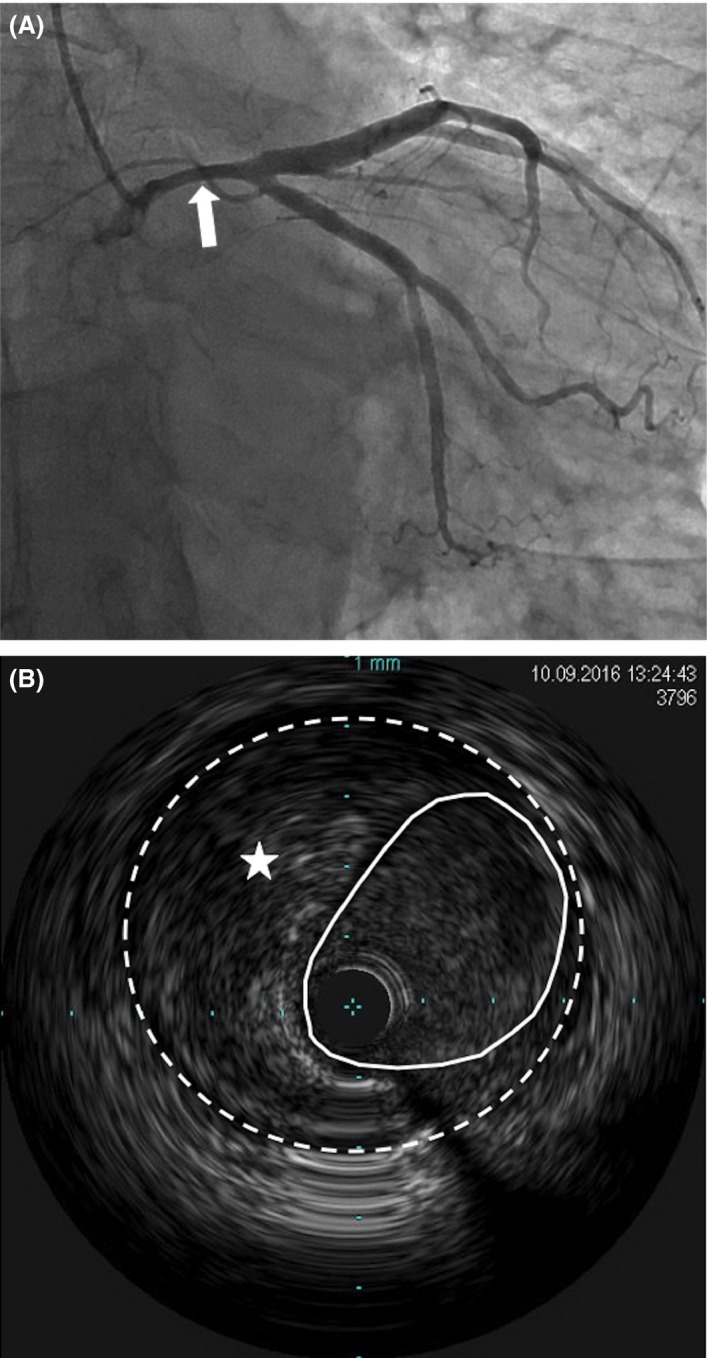
Initial cardiac catheterization and intravascular ultrasound. (A) Caudal view of the left main coronary artery with diffuse loss of vessel diameter (arrow). (B) Left Main intravascular ultrasound exhibiting intramural hematoma (star) with luminal narrowing (dotted line). The solid line represents the adventitia. No obvious dissection flap was identified.

**Figure 2:**
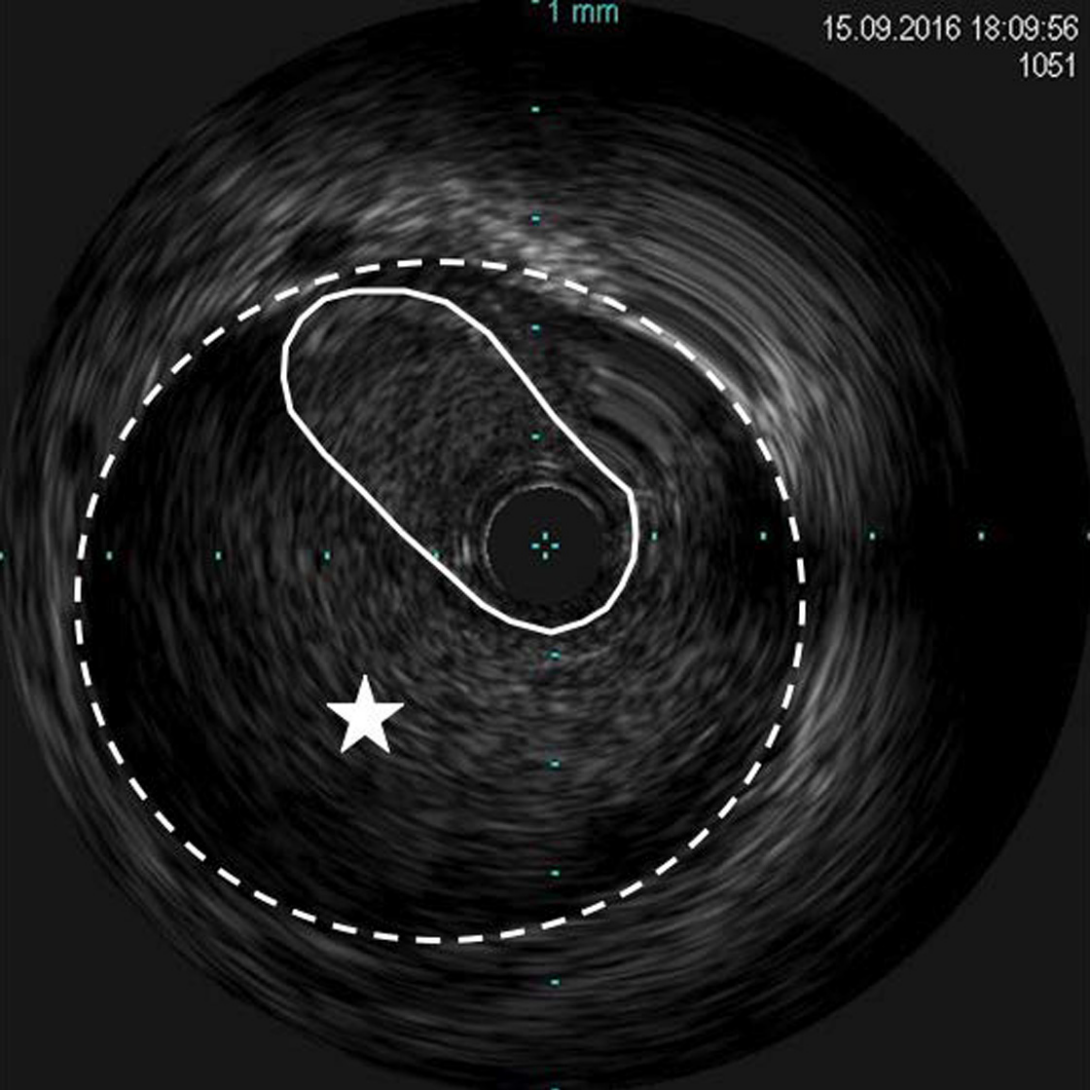
Repeat intravascular ultrasound of the left main coronary artery. The inner region (solid line) denotes the left main lumen. The outer region (dotted line) denotes the adventitia of the left main with evidence of intramural hematoma (star). There is further compromise of the left main lumen.

**Figure 3:**
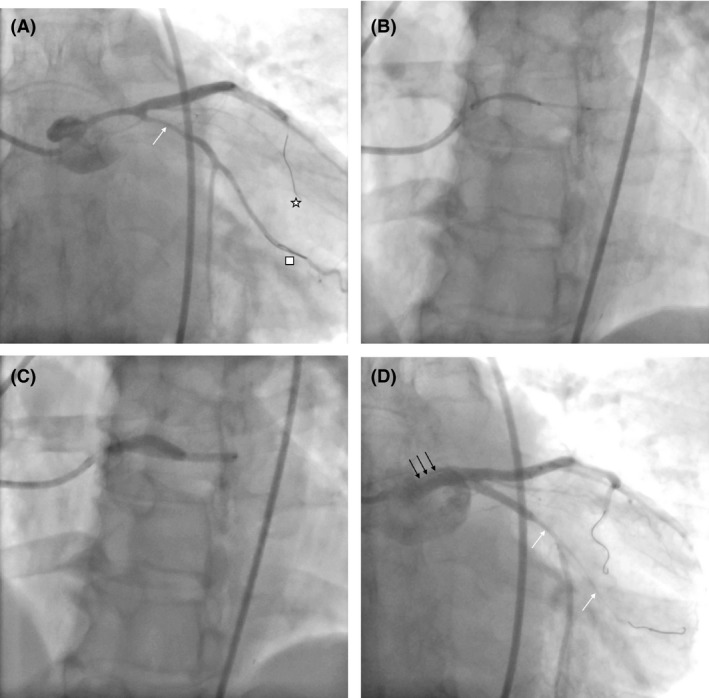
Repeat coronary angiogram. (A) Caudal view of the left coronary tree with angioplasty wires observed down the left anterior descending (star) and circumflex–obtuse marginal arteries (square). Extension of the hematoma is evidenced in the circumflex (arrow). (B) Positioning of two stents (SKS technique) into the left main‐left anterior descending and left main‐circumflex arteries prior to inflation. (C) Simultaneous inflation of both stents with “double‐barreling” in the left main. (D) Caudal angiogram revealing loss of the left circumflex–obtuse marginal circulation after stent deployment (white arrows). Also evidenced in this view is a dissection in the left main (black arrows).

A 51‐year‐old woman with no previous cardiac history and no cardiac risk factors presented to a rural emergency department with severe, nonradiating, substernal chest pain. An electrocardiogram revealed nonspecific ST/T wave changes in the anterolateral leads with a moderately elevated troponin level. The patient was diagnosed and managed as a non‐ST‐segment myocardial infarction (NSTEMI). Initial coronary angiography revealed angiographic evidence of left main (LM) SCAD with a 50% luminal stenosis (Fig. 1A), and intravascular ultrasound (IVUS) confirmed evidence of subintimal hematoma (Fig. 1B). The hematoma was largely restricted to the LM coronary artery. As the patient was asymptomatic, decision was made to manage the patient conservatively with repeat IVUS in 1 week to monitor progression. Repeat IVUS showed evidence of further luminal narrowing in some segments (Fig. 2) with improvements in others. Again, as the patient remained asymptomatic, decision was to continue with conservative management. However, she subsequently developed recurrent ischemic symptoms associated with frequent premature ventricular ectopic beats, and therefore, we decided to proceed with LM PCI. The LM was engaged via a transfemoral approach with a seven French JL4 guiding catheter (Cordis, California, United States), and the left anterior descending (LAD) and left circumflex (LCX) arteries were wired with Balance Middleweight Universal (BMW) wires (Abbott Vascular, Illinois, United States). Repeated contrast injections resulted in worsening hydraulic dissections and propagation of compressive hematoma into the proximal circumflex artery (Fig. 3A).

**Figure 4:**
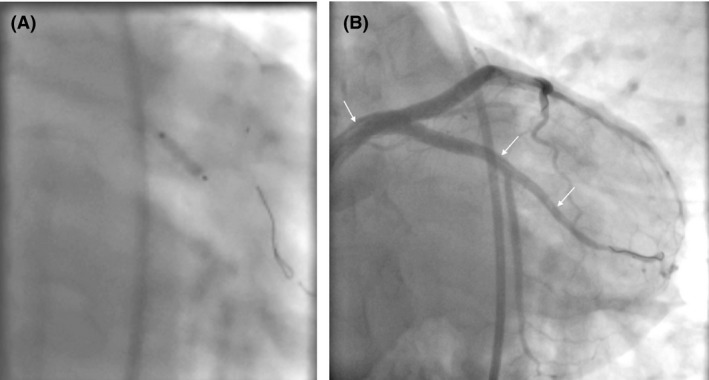
Cutting balloon for management of propagated subintimal hematoma. (A) Angiogram of inflation of the cutting balloon in the left circumflex. (B) Caudal angiogram revealing restored flow down the left circumflex and obtuse marginal vessels with evidence of a dissection in both vessels (arrows).

The decision was made to proceed with PCI of the LM‐LAD‐LCx bifurcation. After a brief deliberation, a simultaneous kissing stent (SKS) technique was chosen as the bifurcation strategy. While not optimal, given the acute worsening of the patient's clinical status, the complexity of the coronary disease (i.e., coronary dissection and subintimal hematoma) and the multistep nature of more evidence‐based bifurcation techniques (e.g., double‐kiss crush or culotte) which would risk rewiring a false lumen, a SKS approach was felt to best balance the risks and benefits in this case. The SKS technique involved deployment of two 4 × 28 mm Xience Xpedition drug‐eluting stents (Abbott Vascular, Illinois, United States) into the LM‐LAD and LM‐LCX (Fig. 3B and C). Following postdilation, there was propagation of compressive hematoma distally into the first obtuse marginal artery associated with only TIMI‐2 flow and there was also evidence of LM dissection plane indicating LM intimal tear as a cause for SCAD (Fig. 3D). The compressive hematoma in the obtuse marginal artery was treated with a 3 × 10 mm Flextome Cutting Balloon (Boston Scientific, Massachusetts, United States) at nominal pressure (Fig. 4A). Following cutting balloon inflation, TIMI‐3 flow was restored in both the circumflex and obtuse marginal arteries (Fig. 4B). There was evidence of type A coronary dissection in the obtuse marginal artery where the cutting balloon was used (Fig. 4B) and no further interventions were performed. The patient was transferred to the CCU where she remained asymptomatic until discharge 2 days later and continues to be asymptomatic at 1‐year follow‐up.

## Discussion

Spontaneous coronary artery dissection can present unique challenges when percutaneous intervention is undertaken. Among these is subintimal hematoma propagation with resultant distal vessel compromise as illustrated in our case. The use of a cutting balloon in the treatment of SCAD has been reported previously 7, 8. The first report by Yumoto and colleagues 7 described performing cutting balloon angioplasty at the site of the SCAD without subsequent stenting. Yumoto and colleagues 7 deployed a cutting balloon to pressures below nominal in the distal and proximal LAD, immediately improving coronary flow, from TIMI flow grade 0–3. Using a similar technique, Alkhouli and associates 8 also reported the use of a cutting balloon in the mid and distal LAD of a patient with SCAD. Similar to the results reported by Yumoto and colleagues 7, coronary flow was restored; however, stenting was performed in the latter case with a good result 8.

We herein describe a novel use of a cutting balloon to treat compressive subintimal hematoma after stenting of LM SCAD. We used a 1:1 balloon‐to‐artery diameter ratio, and inflation was only to nominal pressure. The length of the balloon used was 10 mm to minimize the extent of intimal damage, and only one inflation was required to completely resolve a longer segment of compressive hematoma.

While we and others 7, 8 have achieved positive results with cutting balloon use in patients with SCAD, this technique does come with risks. Although the pathophysiology and etiology of SCAD are not fully elucidated, it has been associated with arteriopathies, inflammatory diseases, coronary spasm, and fibromuscular dysplasia 3, 9, 10, 11. These patients likely possess some as yet unidentified abnormalities in coronary arterial anatomy and/or physiology. The consequences of iatrogenic intimal laceration and trauma in patients with SCAD are not known. There is theoretically an elevated risk of propagation of the coronary dissection or frank vessel rupture. As a result, cutting balloon use in this population should be carried out cautiously in select cases.

In summary, the use of cutting balloons in patients with SCAD requiring angioplasty can be performed safely with resultant improvement in antegrade blood flow in vessels compromised by subintimal hematoma propagation but needs to be considered with caution.

## Authorship

BM: involved in preparing and writing the manuscript. MV: involved in the angioplasty and revision of the manuscript. Both authors approved the final version of the case report for submission to *Clinical Case Reports*.

## Conflict of Interest

BM has no disclosures pertinent to the content of this manuscript. MV received speaking fees and proctoring honoria from Boston Scientific and Abbott Vascular.
